# Revealing Potential Biomarkers of Functional Dyspepsia by Combining ^1^H NMR Metabonomics Techniques and an Integrative Multi-objective Optimization Method

**DOI:** 10.1038/srep18852

**Published:** 2016-01-08

**Authors:** Qiaofeng Wu, Meng Zou, Mingxiao Yang, Siyuan Zhou, Xianzhong Yan, Bo Sun, Yong Wang, Shyang Chang, Yong Tang, Fanrong Liang, Shuguang Yu

**Affiliations:** 1 Acupuncture and Tuina College, Chengdu University of Traditional Chinese Medicine, Chengdu, Sichuan, 610075, China; 2 National Center for Mathematics and Interdisciplinary Sciences, Academy of Mathematics and Systems Science, Chinese Academy of Sciences, Beijing, 100080, China; 3 National Center of Biomedical Analysis, Beijing, 100850, China; 4 Department of Electrical Engineering, National Tsing Hua University, Hsinchu, 300, Taiwan

## Abstract

Metabonomics methods have gradually become important auxiliary tools for screening disease biomarkers. However, recognition of metabolites or potential biomarkers closely related to either particular clinical symptoms or prognosis has been difficult. The current study aims to identify potential biomarkers of functional dyspepsia (FD) by a new strategy that combined hydrogen nuclear magnetic resonance (^1^H NMR)-based metabonomics techniques and an integrative multi-objective optimization (LPIMO) method. First, clinical symptoms of FD were evaluated using the Nepean Dyspepsia Index (NDI), and plasma metabolic profiles were measured by ^1^H NMR. Correlations between the key metabolites and the NDI scores were calculated. Then, LPIMO was developed to identify a multi-biomarker panel by maximizing diagnostic ability and correlation with the NDI score. Finally, a KEGG database search elicited the metabolic pathways in which the potential biomarkers are involved. The results showed that glutamine, alanine, proline, HDL, β-glucose, α-glucose and LDL/VLDL levels were significantly altered in FD patients. Among them, phosphatidycholine (PtdCho) and leucine/isoleucine (Leu/Ile) were positively and negatively correlated with the NDI Symptom Index (NDSI) respectively. Our procedure not only significantly improved the credibility of the biomarkers, but also demonstrated the potential of further explorations and applications to diagnosis and treatment of complex disease.

Functional dyspepsia (FD) is a common gastrointestinal disease accompanied by epigastric pain, nausea, vomiting, abdominal distension, poor appetite, belching and other symptoms but no underlying organic changes in pathology^1^. It poses a challenge to both society and medicine due to its fairly high incidence and poor control rate^2^. Studies have shown that multiple pathological processes are involved in the development of FD, including biological, physiological and psychological factors. However, until now, the objective diagnostic methods for FD in clinical practice remain unreliable. Most patients are diagnosed FD by questionnaires, which largely depends on patients’ personal perceptions and somatic symptoms. In addition, as many functional digestive disorders share similar in symptoms, there is considerable clinical overlap between FD and other functional gastroenterological disorders^3^.

According by Rome III, a system developed to classify functional gastrointestinal disorders(FGIDs) and the most popular diagnostic criteria, FD is a typical systematic and metabolic disease^4^. Metabolic disturbances, such as abnormal plasma levels of hypercalcemia, heavy metals^5^, acylated ghrelin^6^, cholecystokinin (CCK)^7^, serotonin (5-HT)^8^, gastrin and leptin^9^ were found in FD patients. It is noteworthy that psychosocial factors also contribute to the pathogenesis of FD. However, it is well-known that guts and brain are highly correlated via bidirectional communication through neural, hormonal, and plasma pathways^10^. Hence, it is proper and fitting in this paper to concentrate on the important factor of abnormal metabolism in the pathogenesis of FD.

Metabonomics is a novel method for studying biological systems. The technology of metabolomics has been widely applied in searching for novel biomarkers in cardiovascular diseases^11^, neuropsychiatric diseases^12^, cancer^13^, and many other diseases with the goal of more accurate diagnosis and better treatment options. By collecting and analyzing various types of body fluids, metabolites such as urine, serum, feces, and other pathological tissues, metabolomics analyzes all small molecular metabolites by high - throughput techniques such as nuclear magnetic resonance (NMR), mass spectrometry (MS), high - performance liquid chromatography (HPLC), gas chromatography (GC), and chromatograph-mass spectrometry. In a previous study^14^, we utilized ^1^H NMR-based metabonomics techniques to compare the plasma metabolic profiles of six female FD patients with those of healthy control subjects. Due to that small sample size, the reliability and credibility of our previous findings might be limited. Therefore, we enlarged the sample size four fold in the present study, and used principal component analysis (PCA) and partial least square discriminant analysis (PLS-DA) to analyze the key metabolites associated with FD. Then, an integrative multi-objective optimization (LPIMO) model was proposed to reveal multi-biomarker panel. Combination with these three methods, we hope we can discover potential biomarkers of FD in a more robust and consistent way.

## Materials and Methods

### Ethical approval

All procedures were designed according to the Declaration of Helsinki’s ethical principles. The study protocol has already been ethically reviewed and approved by Ethics Review Committee of the 1^st^ Teaching Hospital of Chengdu University of TCM [2007KL-002]. Patients were aware of their involvement and signed a written informed consent agreeing to the use of the resulting information for medical publications.

### Subjects and participants

All participants were recruited from the 1^st^ Teaching Hospital of Chengdu University of Traditional Chinese Medicine. The subjects recruited had at least one of the following symptoms: postprandial discomfort, early satiety, upper abdominal pain or epigastria burning sensation reported to last over 6 months. In addition, no organic diseases were found by ultrasound or other test (including evidence from upper gastrointestinal endoscopy) to explain these symptoms. Patients with esophageal reflux disorder, other functional esophageal disorders, *Helicobacter pylori* infection, or structural lesions or functional abdominal pain identified by gastrointestinal endoscopy and recent available visit record were excluded. In addition, pregnant women were excluded. Healthy subjects with the same age and BMI were recruited for the control group.

### NDI scores and sample collection

A standardized questionnaire based on the Chinese version of the Nepean Dyspepsia Index (NDI), which had been already tested for validity and reliability in a previous study^14^ was filled out by each patient. The NDI scores consist of three parts: a symptom checklist that measures the frequency, intensity, and level of discomfort of 15 upper gastrointestinal symptoms over the prior 14 days; 25 items designed to assess Quality of Life (QoL), and an 11-item questionnaire designed to measure the relevance or importance of the above items. The total score for each symptom on the checklist was calculated by adding its corresponding frequency, severity and level of discomfort. All fasting venous blood samples (about 5 mL) were collected at about 8:00–9:00 am.

### ^1^H NMR experiments

^1^H NMR spectra of the plasma samples were acquired in the same way as our previous study^14^. Briefly, 500 μL plasma was collected from each venous blood sample by centrifugation at 1000 × g at 4 °C for 10 min. Then it was mixed with 300 μL plasma, 250 μL D_2_O and 50 μL 3-trimethylsilyl-^2^H_4_-propionic acid sodium salt (TSP) in D_2_O (1 mg/ml) in a 5 mm NMR tube. ^1^H NMR spectra of the plasma samples were acquired on a Varian INOVA 600 MHz NMR spectrometer at 27 °C. Two pulse sequences were used: First, Carr–Purcell–Meiboom–Gill (CPMG) spin-echo pulse sequences with a total spin–spin relaxation delay (2nτ) of 320 ms were taken. Free induction decays (FIDs) were collected into 32 K data points with a spectral width of 8000 Hz and 64 scans. The FIDs were zero-filled to double size and multiplied by an exponential line-broadening factor of 0.5 Hz prior to Fourier transformation (FT). In addition, diffusion-edited experiments were also carried out with bipolar pulse pair-longitudinal eddy current delay (BPP-LED) pulse sequence^15^,^16^. The gradient amplitude was set at 35.0 G/cm, with a diffusion delay of 100 ms. A total of 128 transients and 16 K data points were collected with a spectral width of 8000 Hz. A line-broadening factor of 1 Hz was applied to FIDs before FT.

### Pattern recognition analysis of NMR data and permutation test

All plasma ^1^H NMR spectra were manually phased and baseline-corrected using VNMR 6.1C software (Varian, Inc.). For CPMG spectra, each spectrum over the range of δ 0.4–4.4 was data-reduced into integrated regions of equal width (0.01 ppm). For BPP-LED data, each spectrum over the range of δ 0.1–6.0 was segmented into regions of equal width (0.01 ppm). The regions containing the resonance from residual water (δ 4.6–5.1) were excluded. The integral values of each spectrum were normalized to a constant sum of all integrals in a spectrum in order to reduce any significant concentration differences between samples^17^,^18^. Identification of metabolites in spectra was accomplished based on information in the Chenomx NMR Suite 5.0 (Chenomx, Calgary, Canada) and the literature.

The resulting integral data were imported into SIMCA-P (version 10.04; Umetrics, Umeå, Sweden) for multivariate analysis. Data were Pareto-scaled prior to analysis. PLS-DA was used to find differential metabolites between groups. The results were visualized by two-dimensional scores plots representing the distribution of samples and the corresponding loadings plots providing information on the contribution of each variable to the pattern in the scores plots. The data were also preprocessed using orthogonal signal correction (OSC) to filter the unrelated variations not correlated to the group membership^19^,^20^.

The permutation test^21^ was performed to test the over-fitting of OSC-PLS after modeling the data. The values of intercepts, R2 and Q2 indicated that whether the model was over-fitted.

### Potential biomarker identification and metabolic pathway analysis

All key metabolites based on OPLS coefficient and variable importance plot (*VIP*) were recognized and identified. Bivariate correlation was used to analyze the correlation between chemical shift of these key metabolites and the NDI scores. After correlation analysis between NDI score and the key metabolites, the physiological and pathophysiological meanings of the potential biomarkers were evaluated using information from the KEGG (Kyoto Encyclopedia of Genes and Genomes) database^22^ and other published references.

### Multi-biomarker panel identification by an integrative multi-objective optimization (LPIMO) model

**Figure 1:**
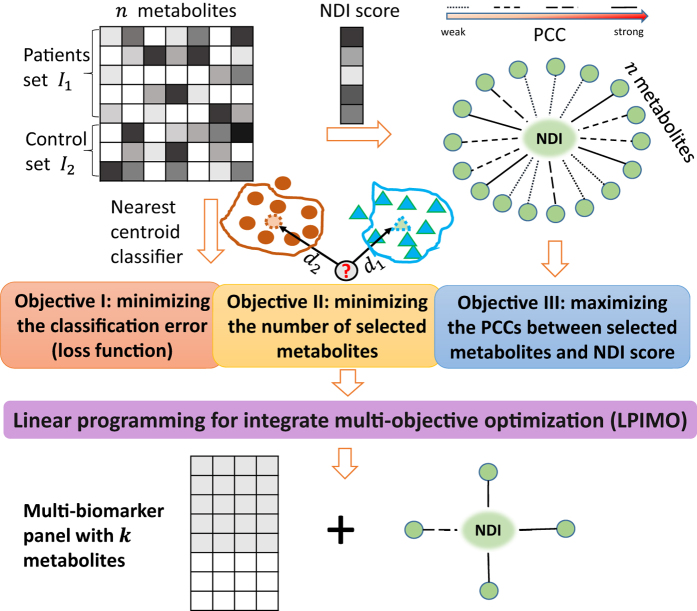
The schematic illustration of LPIMO. The metabolomics data matrix is used for our algorithm and then the correlations of NDI score and PPMs are constructed by calculating the absolute value of Pearson correlation coefficients (PCCs). The nearest centroid classifier is used to distinguish the patient set (brown circles) from the control set (turquoise triangles). The dot circle and triangle denote the centroids of the patients and control set. d_1_ and d_2_ denote the distances between the query sample and control and patients set centroids. Our model classifies a query sample to control set if d_1_ is smaller than d_2_, otherwise, the sample is classified as patients set. Under the nearest centroid classifier, LPIMO minimizes the classification error and the number of selected PPMs, and simultaneously maximizes the PCCs between selected PPMs and NDI score. This leads to a multi-objective optimization problem. To ensure the computation efficiency, a linear programming is used to integrate the multi-objective optimization by introducing weights for each objective. Finally, LPMIO identifies a multi-biomarker panel with good diagnosis ability and high correlation with NDI score.

LPIMO, was proposed to select a small set of PPMs (Chemical shift δ, parts per million (ppm)) which had good diagnostic ability and high correlation with NDI score. The model selected a minimal set of PPMs as biomarkers to distinguish FD patients from controls and simultaneously maximize the pearson correlation coefficient (PCC) between selected PPMs and the NDI score. A schematic illustration of LPIMO is shown in Fig. 1. LPIMO uses three criteria to select a set of PPMs: 1) classification is minimized to attain the best accuracy based on nearest centroid classifier; 2) the number of selected PPMs is minimized to remove redundancy and reduce noise; 3) the PCCs between selected PPMs and the NDI score in FD patients is maximized to select biomarkers correlating well with the NDI score. To balance the three objectives, a multi-objective optimization problem was formulated. After solving the optimization problem, the optimal set of PPMs is multiple biomarker panels.

Suppose ***x*** is the metabolomics data with *m* samples and *n* ppms. The i-th row vector *x*_*i*_ denotes the sample *i* and the j-th column vector *x*_*j*_ denotes the PPM *j*. *y*_*i*_ denotes the NDI score for i-th patient. We introduce *w*_*j*_ ≥ 0, to represent the weight for each PPM. This is the variable to be determined in our model. Then the multiple-biomarker panel identification problem can be formulated as follows:


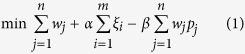


Subject to


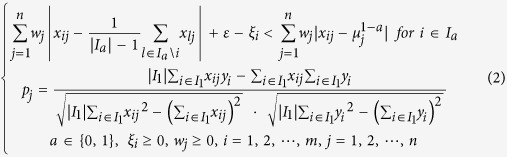


where *ε* is a positive and sufficiently small constant value. *a* is the class label and it takes 1 for patients set and 0 for control set. 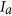 is the set of samples belonging to class *a* and 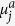 is the average of PPM *j* for 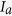. 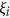 is the slack variable, for the classification error of sample *i*. 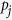 is the PCC between NDI score and PPM *j* in 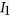.

In the objective function, the first term 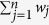 denotes the summarization of weights for those selected PPMs. It is minimized to select a few of PPMs so as to reduce the noise and further enhance the interpretability; the second term 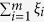 denotes classification error for all samples and it should be minimized to get high accuracy; the third term 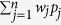 denotes the weighted summarization of PCCs for selected PPMs and it should be maximized. Here *α* and *β* are two parameters to balance the three terms in a single optimization model. The first constraint represents that sample 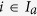 is nearer to the centroid of class *a* than the centroid of the other classes in the training set under the tolerable error 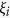. The second constraint is the definition of 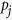 as the PCC between the PPM *j* and the NDI score. The overall goal of this integrative model is to select a minimal set of PPMs that minimize the classification error and simultaneously maximize the weighted summarization of the correlations with NDI score.

We tuned the two parameters *α* and *β* by grid search. For *α*, we tested 1, 10, 100, 1000, and 10 000. For *β*, we tested 0.001, 0.01, 0.1, 1, 10, and 100. Smaller *α* selects fewer PPMs. Larger *β* tends to select PPMs closely associated with the NDI score. We selected the best parameters by balancing the classification error and the weighted summarization of PCCs. By the definition of 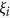, the leave-one-out cross validation (LOOCV) error is  
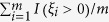
, and used to find optimal parameters.

## Results

### Clinical information and NDI score results

Ultimately 36 eligible female patients were included in this study with mean age 21.4 ± 1.56 (range 21–26) and body mass index 18–24^23^. None of the patients had any organic abnormality according to endoscopy, and other tests including blood sugar, blood chemistry, liver function (ALT, AST), renal function (BUN, Scr), electrocardiogram and abdominal B ultrasound. The NDI symptom scores have an average of 41.74 ± 11.35 and the QoL scores an average of 49.43 ± 7.21. Fifteen healthy females matched for age and body mass index were recruited as controls. They had no other organic disease and their all vital signs were normal. Both patients and healthy subjects had been free of acute illness and did not smoke or drink alcohol, coffee, or tea for 2 weeks before the study, as there is evidence that these factors can interfere with metabolism^24^,^25^,^26^,^27^. All dietary intake and exercise were recorded.

### Metabolomics results

#### ^1^H NMR spectra

**Figure 2:**
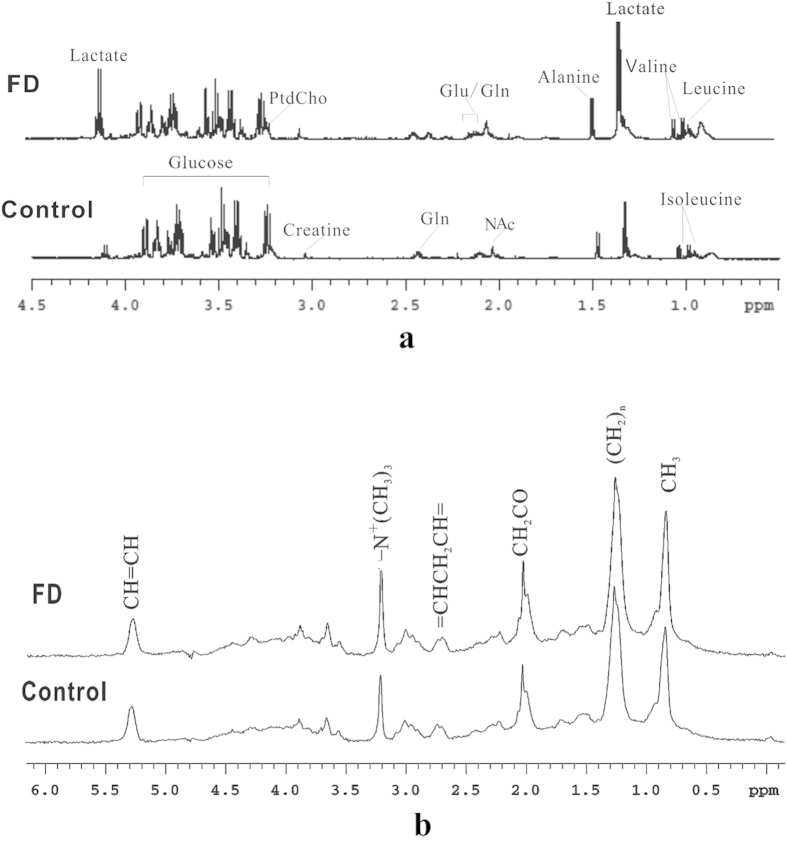
Typical ^1^H NMR spectra. (**a**) Typical ^1^H NMR CPMG spectra of plasma samples from FD patients and controls. NAc, N-acetyl methyl groups of glycoproteins; Gln, glutamine; Glu, glutamate. (**b**) Typical ^1^H NMR LED spectra of plasma samples from FD patients and controls. Main metabolites have been assigned in the spectra.

Plasma comprises both low molecular weight metabolites and high molecular weight protein and lipoproteins. Figure 2a shows the typical low frequency CPMG spectra of plasma samples obtained from FD patients and healthy subjects. Typical LED ^1^H NMR spectra are illustrated in Fig. 2b with the major components identified. From the spectra, we could find that lactate, glucose, alanine, high density lipoprotein (HDL), phosphatidylcholine (Phosphatidycholine, PtdCho) and other metabolites have changed. However, results needed to be confirmed by pattern recognition analysis.

#### Multivariate analysis and potential biomarker discovery

**Figure 3:**
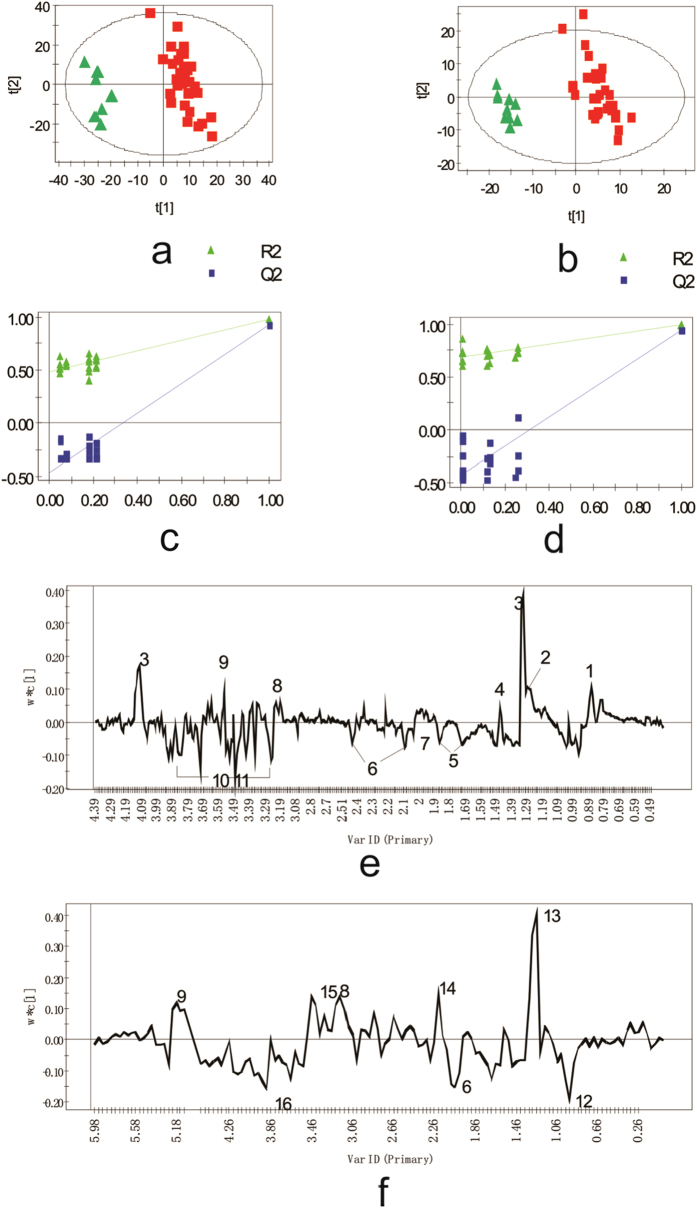
OPLS-DA results of CPMG data (a, c, e) and LED data (b, d, f) of plasma from FD patients and controls, including score plots(a,b), permutation tests, (d) and corresponding loading plots (e,f). The t[1] and t[2] represent the first and second principal components, showing their different distributions due to metabolic differences between samples; w*c[1] represent the corresponding loading to t[1] and t[2] respectively, showing that the corresponding metabolites cause these differences. The eclipse in score plot shows the 95% confidence area of the Hotelling test. Both CPMG score plots (**a**) and LED score plots (**b**) show plasma metabolites of FD patients (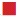)are different from those of control subjects(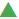). The main changed metabolites are: 1. Lipid CH3; 2. Llipid CH2; 3. Lactate; 4. Analine ;5. Lysine; 6. Glutamine; 7 NAc; 8. PtdCho; 9.Glycerol; 10.Glucose; 11.Proline; 12.Leu/Ile; 13. HDL; 14. Acetoacetate; 15.α-glucose; 16. β-glucose.

Moderate separation could be observed in different groups of the PCA score plots. The OSC-PLS scores plot (Fig. 3a) of the CPMG spectra of the serum samples showed that different groups were clearly separated along PC1 and PC2 with total variation (R2Y) accounting for 95.1% and accumulated Q2 of 90.4%. The permutation tests showed that the intercept for R2 is 0.489 and the intercept for Q2 is −0.46, which indicate that the model is good (Fig. 3c). The score plot (Fig. 3b) of the LED spectra of serum samples showed that PC1 explains the main differences between these two groups. The parameters measured in the LED model showed that PC1 and PC2 accounted for 92.7% of total variance (R2Y), with an accumulated Q2 of 84.4%. The permutation tests showed that the intercept for R2 is 0.687 and the intercept for Q2 is −0.43 which indicates that the model is not over fitted (Fig. 3d).

The corresponding loading plots of CPMG and LED (Fig. 3e,f) identified the metabolites that contributed to these differences. Compared with controls, more than 10 metabolites were altered in FD patients with very important projects (*VIP*) more than 2.0. Levels of HDL (δ0.85, 1.25, 1.26), lactate (δ1.32, 1.33, 4.1, 4.11), PtdCho (δ3.22), Acetoacetate (δ2.22) and LDL/VLDL (δ1.29, 0.91) were higher in the plasma of FD patients. Leu/Ile (δ0.92–0.96, 1.00–1.01, 1.28), proline (δ3.35, 4.12), glutamine (δ2.1) and N-acetylglycosylprotei (NAc) were lower in FD patients. More details are shown in Table 1.

#### Correlations analysis

Correlation analysis was used to identify correlations between full-spectra key metabolites and the NDI scores. The results revealed that though PtdCho is positively correlated with the NDI symptom score (*P* < 0.01), Leu/Ile was negatively correlated (*P* < 0.05). Other metabolites showed no significant correlation with NDI symptom score (Table 2). We further conducted correlation analysis of full-spectra key metabolites with the QoL score in NDI and found no significant correlation (Table 2). Therefore, the results primarily suggested that PtdCho and Leu/Ile in plasma are potential biomarkers of FD.

#### Multi-biomarker panel identification by LPIMO

By LPIMO, we identified a multi-biomarker panel (Table 3) consisting of δ3.22, δ1.87, δ1.72, δ1.7, δ1.69, δ5.02 and δ0.94, and achieved leave-one-out cross validation (LOOCV) accuracy 1.0. Five PPMs in this panel show positive correlation with NDI symptom score (PCC > 0.37) and two show negative correlation (PCC < −0.22). PtdCho (δ3.22) is the most important biomarker in the panel and positively correlated with NDI symptom score (PCC = 0.50). In addition, Leu/Ile (δ1.7, δ0.94) was also selected in the panel as another potential biomarker and was negatively correlated with NDI symptom score.

**Figure 4:**
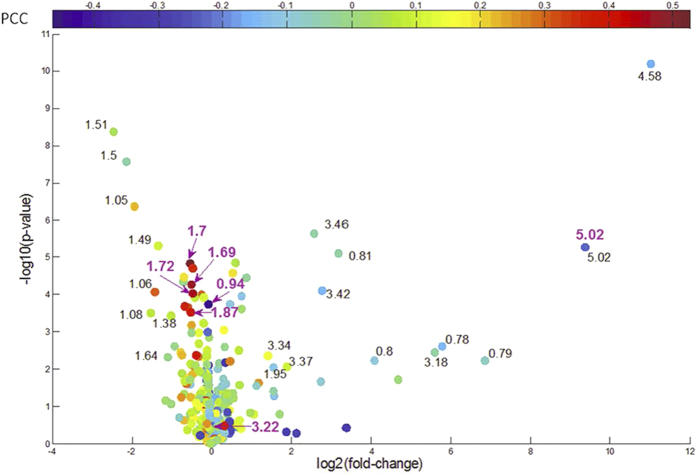
The volcano plot for all PPMs. Each circle denotes a PPM and the color denotes the PCCs between PPM and NDI score. PPMs with black label are significant. (p-value < 0.001 & |log2(fold-change)|>1) in volcano plot analysis. Bold purple PPMs denotes the PPMs identified by LPIMO.

LPIMO also identified some interesting biomarkers, which are presented in a volcano plot^28^. In Fig. 4, the volcano plot was constructed by plotting the negative log of the p-value (usually base 10) on the vertical axis and the log of the fold-change on the horizontal axis (usually base 2). In fact, most PPMs selected by volcano plot (PPMs with black text label) had no-significant correlation with NDI symptom score (Fig. 4). For example, δ4.58 performed best not only by t-test but also had the largest fold-change. There were three PPMs, δ5.02 (PCC = −0.22), δ1.06 (PCC = 0.29) and δ1.05 (PCC = 0.22), for which the absolute value of PCC was more than 0.2. However, LPIMO identified the multi-biomarker panel (bold purple PPMs) that were most strongly correlated with the NDI symptom score (PPC > 0.2). Moreover, LPIMO also identified δ5.02, which performed best among the three PPMs in the volcano plot. In addition, LPIMO identified δ3.22, which showed no-significance in the volcano plot but correlated well with the NDI symptom score (PCC = 0.50).

#### Metabolic pathway analysis of potential biomarkers

According to the KEGG database and other references, several enzymes, including glutathion S-transferases (GSTs), leucine aminopeptidase (LAP), cyclooxygenase (COX), hormone-sensitive lipase (HSL), and fatty acid synthase (FAS) are involved in the biosynthesis and degradation of valine, leucine and isoleucine. Regarding PtdCho, relevant enzymes include phosphocholine transferase, phosphorylcholine-cytidyl- transferase (CTP), lecithin-cholesterol acyltransferase (LCAT), etc. Besides, phosphatidylinositol/phosphatidylcholine transfer proteins-β(PITP-β) is also very important in the glycerophospholipid metabolism.

## Discussion

### The combination of NMR and LPIMO can find more abnormal metabolites and screen potential biomarkers in FD patients

The present study suggests that conventional biochemistry test did not show significant changes in plasmatic blood sugar and blood lipid of FD, while our NMR test revealed significant changes in the levels of glucose, HDL, low density lipoprotein (LDL), very low density lipoprotein (VLDL) and other metabolites in the plasma samples of FD patients. Compared with healthy control, FD patients showed significantly higher PtdCho, HDL, acetoacetate, proline, α-glucose and LDL/VLDL. However, lactate, Leu/Ile, unsaturated fatty acid, glutamine and β-glucose levels were significantly lower in FD patients. Therefore, we obtained more information about the changes in key metabolites by applying NMR than was possible with conventional clinical biochemical tests. As one of the major metabolomic techniques, NMR is intrinsically not as sensitive as mass spectrometry (MS). Its detection limits are in micro to millimolar range, whereas it is common in nanomolar range for MS. Normally one hundred or less of metabolites can be detected by NMR, as compared with hundreds of metabolites detected by MS methods^29^. Therefore, the possible changes of less abundant metabolites may not be detected by NMR. However, most of the NMR detected metabolites can be identified with chemical identity, and cover a range of representative molecular classes, such as amino acids, sugars, organic acids, amine and ketones^30^. In addition, samples detected by NMR need almost no treatment so that the small molecules can be analyzed in a more intact way^29^,^31^. In this point, NMR-based metabonomic analysis can still render potential biomarkers for diseases^32^.

Unlike previous metabonomic studies including our own NMR study^14^, the current study introduced a three - step strategy to identify potential biomarkers of FD. First, the ^1^H NMR method can get a relative full change of the metabolomic spectra of FD. Second, correlation analysis can identify the association between full-spectra key metabolites and clinical disease traits. In addition, we introduced an integrative model to select a multi-biomarker panel by LPIMO. By solving a linear programming problem, we are able to identify a multi-biomarker panel that has good diagnostic capability and correlation with NDI symptom score. Furthermore, we re-correlated the identified metabolites with symptoms of FD. This procedure has generated more detailed and in - depth data for further characterization of metabonomics changes in FD, enabling us to conceive a network of metabolic feature spectra and improving the usefulness and applicability of these markers for clinical diagnosis.

### Leu/Ile and Ptdcho have been identified as potential biomarkers of FD with repeated validation

Our study, for the first time, demonstrated that lower levels of Leucine/isoleucine and higher levels of phosphatidycholine were closely associated with FD symptoms in young females, indicating that Leu/Ile and Ptdcho are potential biomarkers of FD. As isomeride, both leucine and isoleucine are essential amino acids and cannot be biosynthesized by the human body itself but must be absorbed from exterior sources. Lower level of leucine and isoleucine reflect malabsorption of necessary amino acid in FD female patients. The important roles of leucine include activating the protein biosynthesis pathway and regulating protein transportation in skeletal muscle and heart. In addition, Leu/Ile also works jointly with valine in various biological reactions and is necessary in the biosynthesis of reduced glutathione hormone (GSH). Abnormal metabolism of Leucine and isoleucine has been found in other digestive system disorder, such as pancreatic cancer^33^ and hepatocellular carcinoma^34^. In addition, leucine and isoleucine are also branch-chain amino acids (BCAAs), which play crucial roles in reducing central fatigue. Higher BCAAs concentration in plasma will reduce the uptake of tryptophan by the brain as well as 5-HT synthesis, thereby delaying fatigue^35^, partially explaining why FD patients often feel tired. Furthermore, reduction of leucine may inhibit the bioactivity of mammalian target of rapamycin (mTOR) in the protein biosynthesis pathway^36^. Lower level of leucine may consequently inhibit the signaling of mTOR and result in the reduction of synthesis of skeletal myoprotein and make FD patients subject to weight loss and skeletal muscle atrophy. In some studies of obese subjects, abnormal plasma concentrations of BCAAs and protein levels of BCAA-catabolizing enzymes in visceral adipose indicate that BCAAs are closely related to adiposity balance, which is also important in FD patients^37^.

Ptdcho is important for cell regeneration and proper functioning of the vital organs of the nervous, blood circulation and immune systems^38^,^39^. It also accounts for more than 70% of total phospholipids within the intestinal mucus layer^40^. In the current study, higher Ptdcho was found in FD patients’ plasma, compared to controls, which conflicts with the results from ulcerative colitis (UC) studies. In UC studies, low mucus Ptdcho is a key pathogenetic factor^41^. Treatment with modified release Ptdcho could improve the impaired barrier function and help relieve intestinal inflammation in UC patients^42^. In fact, discrepancy in Ptdcho level may indicate the existence of different types of metabolic- related pathogenesis in different gastrointestinal diseases. For instance, FD patients with higher levels of Ptdcho may have higher Cholecystokinin (CCK)^43^. CCK can inhibit gastric motility and emptying via a capsaicin sensitive vagal pathway that is involved in the regulation of food intake. It also involves in the pathogenesis of panic disorder, anxiety and pain in FD. Therefore, increasing level of Ptdcho may relate to the FD symptoms through CCK pathway. In addition, CDP-choline, the substrate of Ptdcho synthesis, is essential to HPA axis. As FD is a mind-body disease, the deregulation of the HPA axis is also very important and therefore further suggests that Ptdcho might be involved in the development of FD.

There are some limitations in our study. For example, FD has two subcategories: postprandial distress syndrome and epigastric pain syndrome. We haven’t analyzed the difference of these two subcategories. Nevertheless, our study provides a systematic resolution to clinical metabonomic data analyses, which not only correlate the clinic characters of the disease, but also take advantage of data analysis. This strategy may contribute to understanding of the mechanisms and accurate diagnosis of a variety of diseases.

## Additional Information

**How to cite this article**: Wu, Q. *et al*. Revealing Potential Biomarkers of Functional Dyspepsia by Combining 1H NMR Metabonomics Techniques and an Integrative Multi-objective Optimization Method. *Sci. Rep.* **6**, 18852; doi: 10.1038/srep18852 (2016).

## Supplementary Material

Supplementary Information

## Figures and Tables

**Table 1:** Plasma metabolites changed in FD patients comparing with control subjects.

Metabolites	Chemical Shift(δ)	FD*vs.*Control	
		Content	*P*
PtdCho	3.21, 3.22	↑	0.04
Alanine	1.46, 1.47, 3.77	↑	0.369
Pro	3.35	↓	0.011
Leu/Ile	0.94,0.96,0.97, 0.99	↓	0.004
Lysine	1.48	↓	0.569
LDL/VLDL	1.3	↑	0.088
Lac	1.34	↑	0.009
Gln	2.09, 2.1	↓	0.015
β-glucose	3.52	↓	0.000
Acetoacetate	2.22	↑	0.384
α-glucose	3.39, 3.73	↑	0.524
HDL	0.86, 1.26	↑	0.004
Choline(NCH_2_)	3.66	↑	0.269

Note: ↑means the level of the metabolites increased;↓means the level of the metabolites decreased.

**Table 2:** Correlation analysis of metabolites obviously changed in FD patients and the NDI symptom score, Quality of Life (QOL) score.

	Lactate	Leu/Ile	NAc	UFA	LDL/VLDL	Gln	HDL	PtdCho	Acetate	Proline	AcAcOH	Alanine	Lysine	Glucose
NDI Symptom Score	r = 0.02	r = −0.44	r = −0.07	r = −0.14	r = −0.04	r = −0.05	r = 0.04	r = 0.50	r = 0.11	r = −0.12	r = 0.07	r = 0.20	r = −0.09	r = 0.01
	*P* = 0.89	*P* = 0.02*	*P* = 0.67	*P* = 0.43	*P* = 0.80	*P* = 0.79	*P* = 0.80	*P* = 0.00**	*P* = 0.54	*P* = 0.49	*P* = 0.70	*P* = 0.27	*P* = 0.61	*P* = 0.94
NDI QOL sore	r = −0.034	r = −0.024	r = −0.035	r = −0.03	r = −0.095	r = −0.024	r = 0.081	r = 0.098	r = 0.174	r = 0.105	r = 0.044	r = 0.36	r = −0.11	r = 0.056
	*P* = 0.842	*P* = 0.887	*P* = 0.837	*P* = 0.868	*P* = 0.581	*P* = 0.89	*P* = 0.64	*P* = 0.57	*P* = 0.311	*P* = 0.543	*P* = 0.800	*P* = 0.84	*P* = 0.35	*P* = 0.743

^*^P < 0.05, **P < 0.01.

**Table 3:** Multi-biomarker panel identified by LPIMO.

Biomarker name	PPM	coefficients derived by LPIMO	NDI symptom score (PCC)	NDI life quality score (PCC)	Fold-change	*P*-value
PtdCho	3.22	3.5115	0.5031	−0.1005	1.0598	0.35713
	1.87	0.9134	0.3732	−0.1660	0.6902	0.0003
	1.72	1.8175	0.4628	−0.1678	0.7185	9.23E-05
Leu/Ile	1.7	0.3670	0.5264	0.0062	0.6874	1.45E-05
	1.69	0.1113	0.4724	−0.0353	0.7010	5.24E-05
glucose	5.02	1.4685	−0.2206	0.0230	Inf	5.17E-06
Leu/Ile	0.94	0.5781	−0.4378	0.0061	0.9411	0.00018
