# Supplementary material for: Revealing Potential Biomarkers of Functional Dyspepsia by Combining 1H NMR Metabonomics Techniques and an Integrative Multi-objective Optimization Method
Source: Sci Rep. 2016 Jan 8;6:18852. doi: 10.1038/srep18852 (PMC4705523; doi:10.1038/srep18852)
Supplement: Supplementary Information [file srep18852-s1.pdf]

Title Page

Revealing Potential Biomarkers of Functional Dyspepsia by Combining ^{1}H NMR Metabonomics Techniques and an Integrative Multi-objective Optimization Method

Authors: *Qiaofeng Wu[1,a], Meng Zou[1,b], Mingxiao Yang[a], Siyuan Zhou[a], Xianzhong Yan[c], Bo Sun[c], Yong Wang[b], Shyang Chang[d], Yong Tang[a], Fanrong Liang[a*], Shuguang Yu[a*]*

a. Acupuncture and Tuina College, Chengdu University of Traditional Chinese Medicine, Chengdu, Sichuan, 610075, China;

b. National Center for Mathematics and Interdisciplinary Sciences, Academy of Mathematics and Systems Science, Chinese Academy of Sciences, Beijing, 100080, China;

c. National Center of Biomedical Analysis, Beijing, 100850, China;

d. Department of Electrical Engineering, National Tsing Hua University, Hsinchu, 300, Taiwan;

Figure legend of supplementary

A stack plot of ^{1}H NMR spectra from FD patients and controls. Main metabolites has been designed in the spectrum.

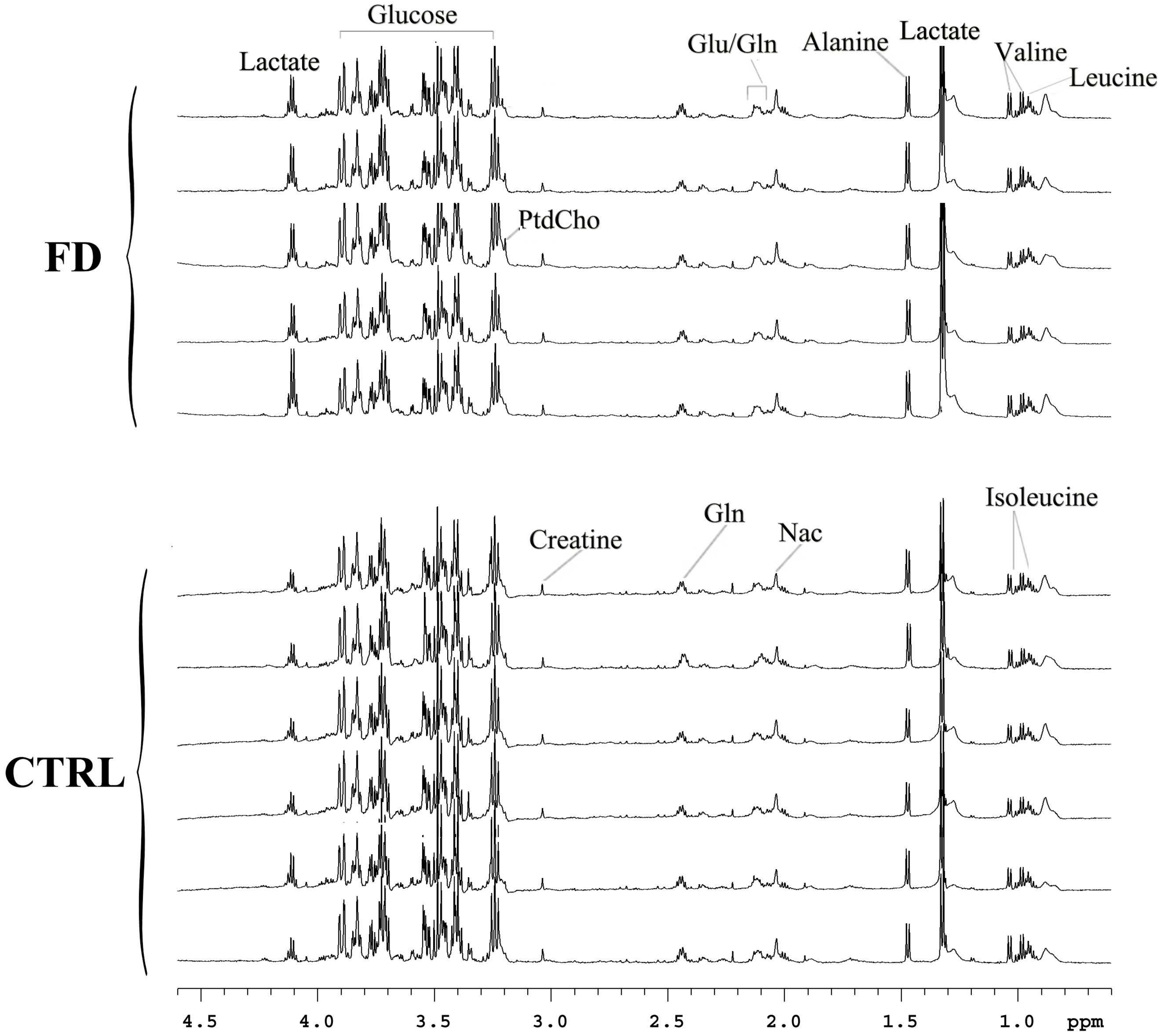

CPMG Spectra

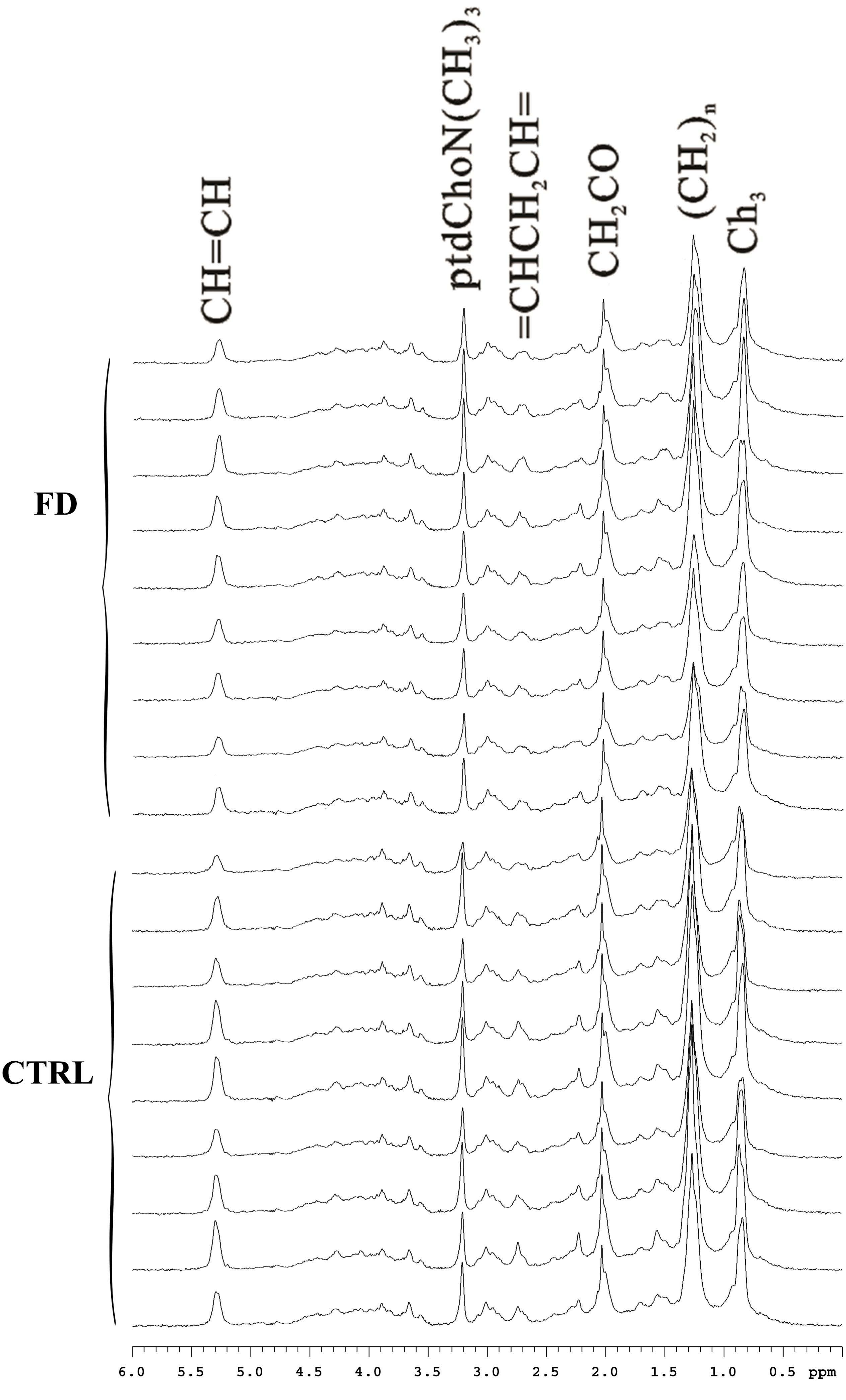
CH=CH
ptdChoN(CH3)3
=CHCH2CH=
CH2CO
(CH2)n
Ch3
FD
CTRL
6.0 5.5 5.0 4.5 4.0 3.5 3.0 2.5 2.0 1.5 1.0 0.5 ppm

LED Spectra
